# Supplementary material for: Combination of Small Extracellular Vesicle-Derived Annexin A2 Protein and mRNA as a Potential Predictive Biomarker for Chemotherapy Responsiveness in Aggressive Triple-Negative Breast Cancer
Source: Cancers (Basel). 2022 Dec 29;15(1):212. doi: 10.3390/cancers15010212 (PMC9818227; doi:10.3390/cancers15010212)
Supplement: Supplementary file 1 [file cancers-15-00212-s001.zip › Supplementary Figure S1.pdf]

Figure S1

A

i) ii) iii)

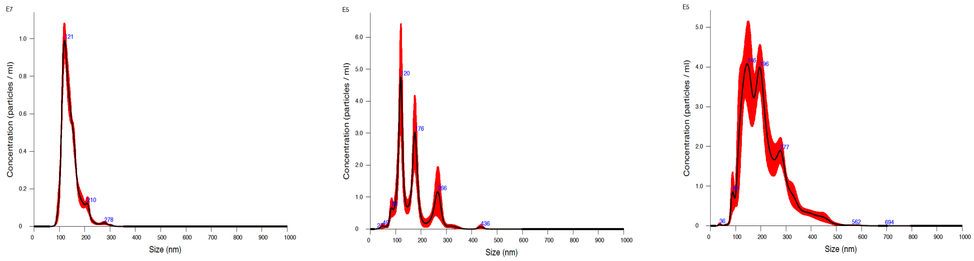

B

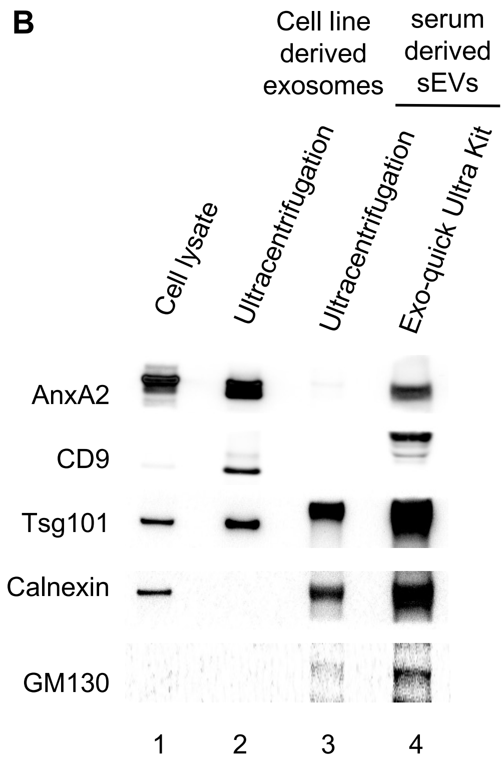

Supplementary Figure S1: Small extracellular vesicles (sEVs) isolation from the mouse serum without IgG pretreatment had increased levels of organelle contaminants. A. NTA analysis of i) Cell supernatant derived sEVs separated by the ultracentrifugation method ii) Mouse serum sample derived sEVs separated by ultracentrifugation method without IgG depletion and iii) Mouse serum sample derived sEVs separated by the ExoQuick Ultra kit without IgG separation. B. Western blot analysis shows the expression of proteins viz. AnxA2, Tsg101, and CD9 as the positive markers; Calnexin (Endoplasmic reticulum marker) and GM130 (Golgi body marker) as the markers for other organelles in the lysates containing an equal number of sEVs loaded in lane 3 and 4. Lane 1 with cell lysate and lane 2 with MDA-MB-231 cell line-derived sEV lysate were used as controls.
