# Supplementary material for: Combination of Small Extracellular Vesicle-Derived Annexin A2 Protein and mRNA as a Potential Predictive Biomarker for Chemotherapy Responsiveness in Aggressive Triple-Negative Breast Cancer
Source: Cancers (Basel). 2022 Dec 29;15(1):212. doi: 10.3390/cancers15010212 (PMC9818227; doi:10.3390/cancers15010212)
Supplement: Supplementary file 1 [file cancers-15-00212-s001.zip › Supplementary Figure S2.pdf]

Figure S2:

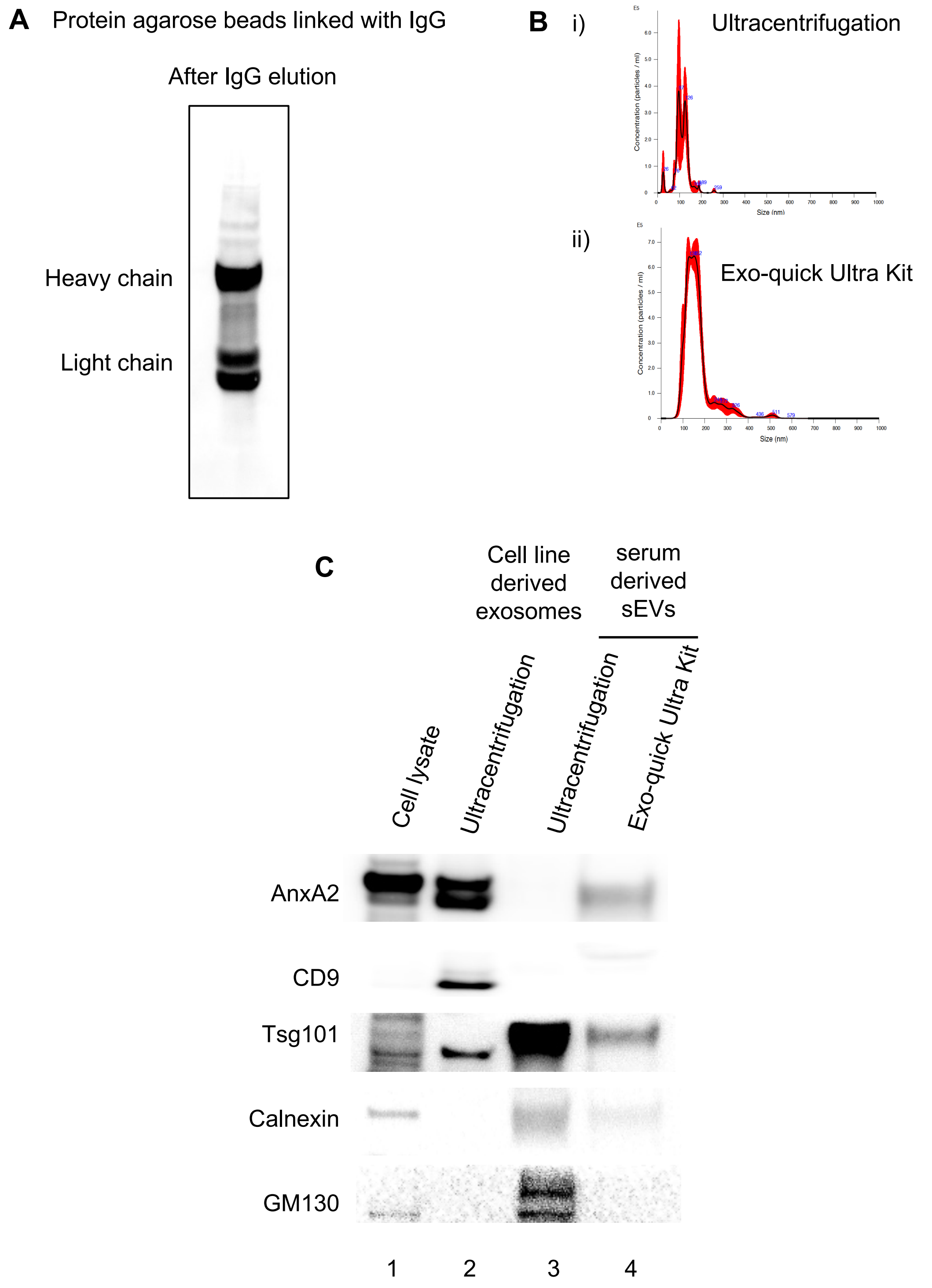

Supplementary Figure S2: Comparison of sEV isolation from serum by Ultracentrifugation and the ExoQuick Ultra kit after IgG separation to reduce organelle contamination. A. Western blot confirming the successful IgG separation from the serum sample using proteinase A/G beads shown as light chain band at 25 kDa and heavy chain band at 50 kDa. B. NTA analysis of the i) Mouse serum sample derived sEVs separated by the ultracentrifugation method with IgG separation and ii) mouse serum sample derived sEVs separated by the ExoQuick Ultra kit with IgG separation. C. Western blot analysis shows the expression of proteins like AnxA2, Tsg101, and CD9 as the positive markers; Calnexin and GM130 as the other organelle markers in lysates containing an equal number of sEVs in lanes 3 and 4. Lanes 1 and 2 showing cell lysate and cell line-derived sEV lysate, respectively, as controls.
