# Supplementary material for: Combination of Small Extracellular Vesicle-Derived Annexin A2 Protein and mRNA as a Potential Predictive Biomarker for Chemotherapy Responsiveness in Aggressive Triple-Negative Breast Cancer
Source: Cancers (Basel). 2022 Dec 29;15(1):212. doi: 10.3390/cancers15010212 (PMC9818227; doi:10.3390/cancers15010212)
Supplement: Supplementary file 1 [file cancers-15-00212-s001.zip › Supplementary Figure S3.pdf]

Figure S3:

IgG depletion from serum samples

200 µl TNBC / normal serum (NS) sample + 50 µl PBS + 10 µl proteinase A/G beads

↓

Incubate for 2 hours at 4^0C

↓

Centrifuge at 3000g for 5 minutes and pellet down the beads

↓

Resuspend the IgG-linked beads in the RIPA buffer

↓

Dilute the sample 1:20 and load on the SDS-PAGE gel

↓

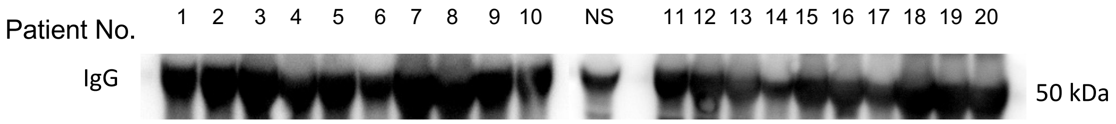

Supplementary Figure S3: IgG depletion by using protein A/G beads and its confirmation. Western blot analysis of the protein bead plus IgG linked to it was done by heating the beads in a boiling water bath. The samples were subjected to the SDS-PAGE. Patient samples no 1-20 were confirmed for IgG elution by staining with anti-human IgG antibody.
