# Supplementary material for: Combination of Small Extracellular Vesicle-Derived Annexin A2 Protein and mRNA as a Potential Predictive Biomarker for Chemotherapy Responsiveness in Aggressive Triple-Negative Breast Cancer
Source: Cancers (Basel). 2022 Dec 29;15(1):212. doi: 10.3390/cancers15010212 (PMC9818227; doi:10.3390/cancers15010212)
Supplement: Supplementary file 1 [file cancers-15-00212-s001.zip › Supplementary Figure S4.pdf]

**Figure S4:**

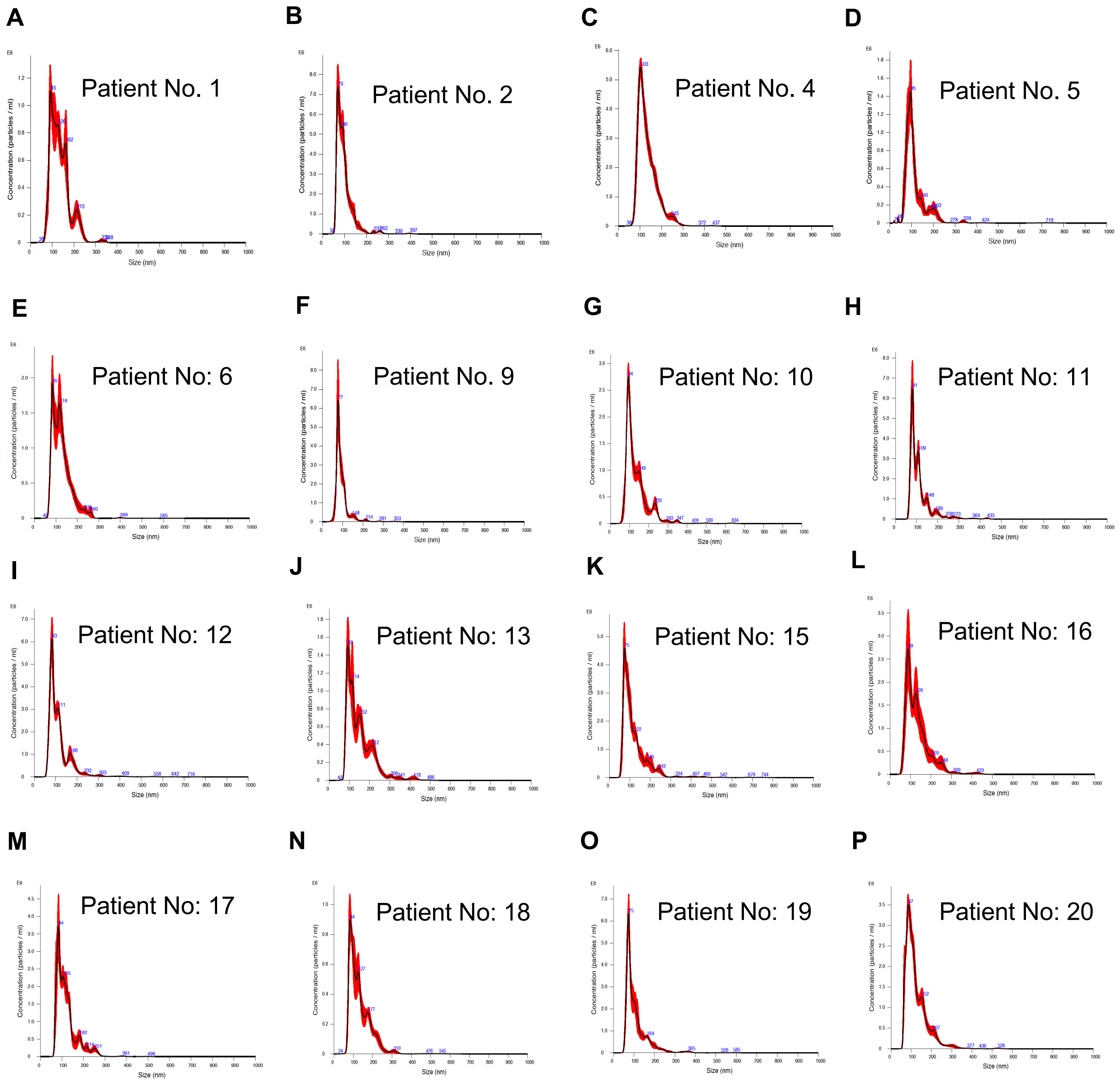


**Supplementary figure S4**: NTA analysis of the sEVs derived from TNBC patients. Patients are denoted as A-P with their patient numbers.
