# Supplementary material for: Combination of Small Extracellular Vesicle-Derived Annexin A2 Protein and mRNA as a Potential Predictive Biomarker for Chemotherapy Responsiveness in Aggressive Triple-Negative Breast Cancer
Source: Cancers (Basel). 2022 Dec 29;15(1):212. doi: 10.3390/cancers15010212 (PMC9818227; doi:10.3390/cancers15010212)
Supplement: Supplementary file 1 [file cancers-15-00212-s001.zip › Supplementary Table.pdf]

Supplementary Table S1: The sizes and concentrations of the sEVs isolated from different human TNBC patient serum samples using the ExoQuick Ultra kit. The sEVs derived from 3 normal serum samples and 17 TNBC patient serum samples are as follows:

Supplementary Table S1 :

Patient No.	Clinical Prognostic stage	sEV size (nm)	No. of sEVs x 10^8 (particles/ml)
1	-	105.5 +/- 7.2	(0.896 +/- 0.0214)
2	-	79.2 +/- 5.2	(3.5 +/- 0.177)
3	-	85.7 +/- 2.2	(3.23 +/- 0.097)
4	1B	87.8 +/- 3.4	(1.86 +/- 0.155)
5	1B	89.5 +/- 4.8	(0.752 +/- 0.0462)
6	1B	94.8 +/- 7.9	(1.37 +/- 0.0517)
7	1B	87.1 +/- 5.3	(2.44 +/- 0.185)
8	2A	93.0 +/- 2.8	(3.89 +/- 0.177)
9	2B	81.0 +/- 3.0	(1.98 +/- 0.165)
10	2B	93 +/- 1.5	(1.6 +/- 0.1)
11	2B	83 +/- 3.9	(2.6 +/- 0.1)
12	3B	81 +/- 1.1	(3 +/- 0.2)
13	3B	77 +/- 3.9	(2.1 +/- 0.1)
14	3B	81 +/- 3.0	(3.7 +/- 0.2)
15	3B	88 +/- 4.3	(2.4 +/- 0.2)
16	3B	106.0 +/- 13.3	(2.07 +/- 0.117)
17	3B	86.0 +/- 5.6	(2.11 +/- 0.0929)

18	3C	89 +/- 8.5	(0.651 +/- 0.02)
19	3C	78 +/- 5.0	(2.8 +/- 0.2)
20	4	84 +/- 5.2	(2.6 +/- 0.1)
