# Supplementary figures and images for: Combination of Small Extracellular Vesicle-Derived Annexin A2 Protein and mRNA as a Potential Predictive Biomarker for Chemotherapy Responsiveness in Aggressive Triple-Negative Breast Cancer
Source: Cancers (Basel). 2022 Dec 29;15(1):212. doi: 10.3390/cancers15010212 (PMC9818227; doi:10.3390/cancers15010212)

Figure S5:

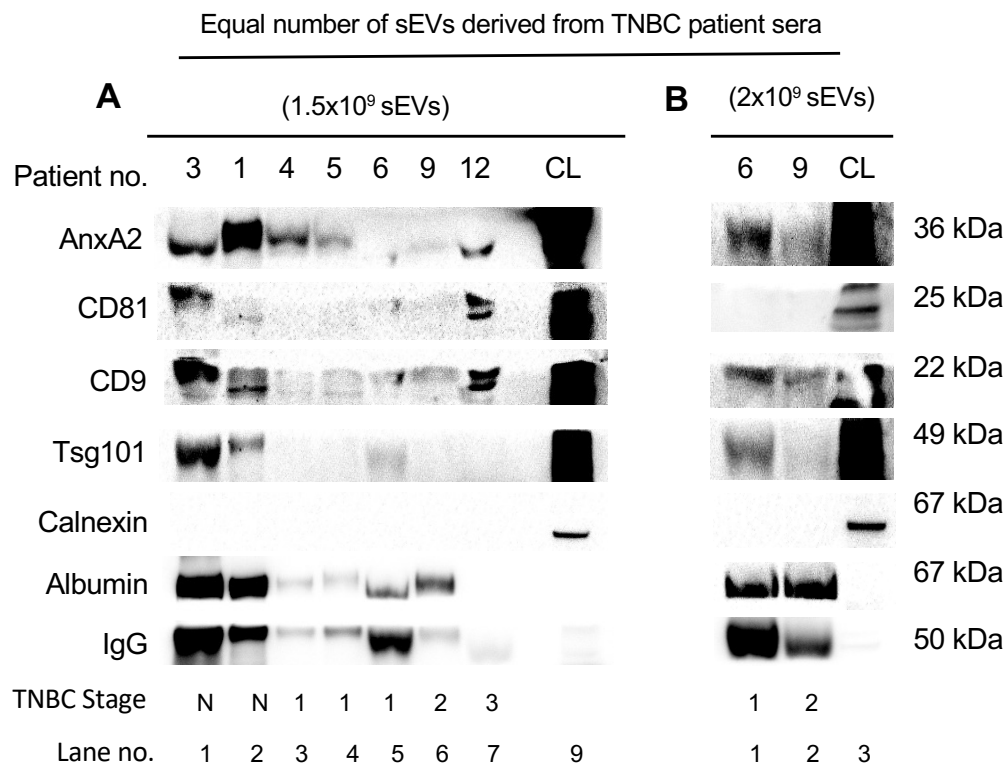

Figure S5: Patient sample derived sEVs bearing AnxA2 and other EV markers.

Supplement: Supplementary file 1 [file cancers-15-00212-s001.zip › Supplementary Figure S5.pdf]
